# A Crabbit Old Woman Wrote This

**Published:** 1983

**Authors:** 


### A CRABBIT OLD WOMAN WROTE THIS

What do you see nurses, what do you see?
What are you thinking when you are looking at me?
A crabbit old woman, not very wise,
Uncertain of habit, with far-away eyes,
Who dribbles her food and makes no reply
When you say in a loud voice – 'I do wish you'd try'.
Who seems not to notice the things that you do.
And forever is losing a stocking or shoe.
Who unresisting or not, lets you do as you will,
With bathing and feeding, the long day to fill.
Is that what you are thinking, is that what you see?
Then open your eyes nurse, you're not looking at me.
I'll tell you who I am as I sit here so still;
As I use at your bidding, as I eat at your will,
I'm a small child of ten with a father and mother.
Brothers and sisters, who loved one another.
A young girl of sixteen with wings on her feet.
Dreaming that soon now a lover she'll meet;
A bride soon at twenty – my heart gives a leap,
Remembering the vows that I promised to keep:
At twenty-five now I have young of my own,
Who need me to build a secure, happy home:
A woman of thirty, my young now grow fast.
Bound to each other with ties that should last:
At forty, my young sons have grown and are gone,
But my man's beside me to see I don't mourn:
At fifty once more babies play around my knee,
Again we know children, my loved ones and me.
Dark days are upon me, my husband is dead,
I look at the future, I shudder with dread,
For my young are all rearing young of their own,
And I think of the years and the love that I've known.
I'm an old woman now and nature is cruel –
'Tis her jest to make old age look like a fool.
The body it crumbles, grace and vigour depart,
There is now a stone where I once had a heart;
But inside this old carcase a young girl still dwells,
And now and again my battered heart swells,
I remember the joys, I remember the pain,
And I'm loving and living life over again,
I think of the years all too few – gone too fast,
And accept the stark fact that nothing can last.
So open your eyes, nurses, open and see
Not a crabbit old woman, look closer – see ME!

---

It appeared when an old lady died in the geriatric ward of Ashludie Hospital, near Dundee, that she had left nothing of any value; then the nurse going through her possessions found a poem. The quality of this so impressed the staff that copies were duplicated and distributed to every nurse in the hospital.

